# U. S. Army Veterinarians

**Published:** 1886-10

**Authors:** James A. Waugh

**Affiliations:** 6th Cav., U. S. A.


### Art. XXII.—U. S. ARMY VETERINARIANS.

BY JAMES A. WAUGH, V. S., 6TH CAV., U. S. A.

Much has been written deploring the low social and professional status of veterinarians employed as veterinary surgeons in the cavalry regiments of the U. S. Army. However, little or nothing has been written in favor of the subject, except some articles advocating the establishment and organization of a Veterinary Department in the U. S. Army, similar to those established and maintained by the war departments of Great Britain, France, Germany, and other European governments. Therefore it may not be amiss for an experienced army veterinarian to indulge in some remarks on the subject.

It is well known that previous to 1879 anyone was considered eligible for appointment to the position of veterinary surgeon in any of the cavalry regiments of the U. S. Army, and usually some superannuated soldier received the appointment to fill a vacancy, merely as a sinecure on account of having rendered faithful services as a family servant, or in military parlance—"a striker" or "a dog-robber." It seems the possession of a little intelligence, with some knowledge and a veterinary education, were not considered essential attainments or requisites necessary to qualify an applicant for appointment to the position of veterinary surgeon in a cavalry regiment of the U. S. Army. In 1879 Army Regulations were amended so as to read:—" Par. 287.—Appointments as Veterinary Surgeons will be confined to the graduates of established and reputable veterinary schools and colleges. They will be appointed by the Secretary of War, in numbers not to exceed the legal establishment, and only on recommendation from the commanding officer of the regiment, supported by the requisite proofs of learning and skill, and by approval of intermediate commanders." [G. O.—36,1879.]

Nevertheless it appears the above has in some cases proved a dead letter, because the non-graduates, or charlatans, empirics and quacks were retained as veterinary surgeons in the cavalry regiments of the U. S. A., and at present the best paying positions are in the hands of quacks and empirics, including the position of "Inspector of cavalry horses for the Division of the Missouri," "the latter resulting in the purchase of anatomically unsound and physically unfit animals in large numbers." Cavalry horses are usually purchased without being subjected to any qualified veterinary examination previous to purchase.

The professional duties of a veterinarian in the U. S. A. are by no means onerous or disagreeable, and he always has farriers to nurse and attend sick or disabled animals. He has plenty of leisure time to study not only his profession but other subjects as well. In some cases he is compelled to comply with special orders to visit all the stables or corrals at certain hours each day, while others are permitted to exercise their own discretion in regard to visiting the stables or corrals. They are supposed to instruct farriers and horse-shoers in regard to the proper performance of their duties, but such is usually only a matter of form. Troop commanders, farriers and horse-shoers generally manage such affairs to suit their own peculiar fancies. Veterinarians are usually stationed at regimental headquarters, and are sometimes taken out with the troops on duty in the field. They invariably move when their regiments change stations. The veterinarian is not permanently stationed at any particular post, but is occasionally ordered to other posts to treat outbreaks of disease, such as strangles, and at the same time instruct the farriers and horse-shoers. They are supposed to treat quartermasters' mules without getting any extra pay for it. Veterinarians are generally allowed to practice all they can on the outside, in private practice, among live stock owned by citizens.

The 1st, 2d, 3d, 4th, 5th and 6th regiments of U. S. cavalry are each legally entitled to one veterinary surgeon, who receives a salary of $75 a month. The 7th, 8th, 9th and 10th regiments are each legally entitled to two veterinary surgeons: one senior veterinary surgeon, who receives $100 a month, and one junior veterinary surgeon, who receives $75. Three of those senior positions are held by quacks, while the junior positions are held by regular graduates. It seems very strange that the authorities continue to foster empiricism and quackery, but decline to decently recognize regularly educated and graduated veterinarians. They get transportation and traveling expenses not to exceed four dollars per day while traveling under special orders.

There is no established or prescribed professional status for veterinary surgeons in the U. S. A., and in some garrisons they are treated as soldiers or teamsters, while at other garrisons they are treated as members of a learned profession; in short, there appears to be some diversity of opinion in the U. S. A. as to whether veterinary surgeons are professional men or common horse grooms, and of course that frequently occasions some wrangling and a little ill feeling, especially if the veterinarian is a regular graduate of some veterinary college or school. However, by a recent ruling of the Lieutenant-General, it has been decided that "veterinary surgeons are considered citizens hired to treat public animals, and in no case are they to be treated as soldiers."

Notwithstanding, one or more veterinary surgeons in the Cavalry regiments are compelled to wear the uniform of enlisted men, salute officers according to military tactics, attend "stables" and groom horses same as any cavalry soldiers do, also attend drills, and muster as if they were enlisted soldiers. In some cavalry regiments the veterinary surgeons are treated as civilians and contract surgeons, and in such cases they usually associate with the officers and their families, or with the better class of citizens and settlers on the frontier. It is very sad, though true, that some young veterinarians in the U. S. A. soon become completely discouraged, then lose self-esteem, finally acquire vicious habits, and are ultimately ruined in every respect.

Practically, the social condition of veterinarians depends entirely on their characters, manner of conduct, habits, etc. They are supposed to have the relative rank of a sergeant-major (an enlisted man), and are usually assigned to quarters in "laundress-row" along with the wives and families of soldiers. These conditions are by no means enticing or flattering to the young veterinarian in the "service of his country," but, fortunately, as a rule, he has not been "reared in the lap of luxury," and of course he only came into the army to earn some money, which will some day enable him to establish a private practice in civil life. There are occasional exceptions to the above rule, and some veterinarians enter the U. S. A., on account of indolence or intemperate habits.

There is no provision for longevity pay, retirement or pension for any veterinarian who is employed in the U. S. A., though contract surgeons employed in the U. S. A. Medical Department, are entitled to all the rights and privileges of retirements, pensions, etc., the same as regularly commissioned officers of the U. S. A.

There are five regiments of artillery in the U. S. A., but there never has been any legal provision for the employment of veterinarians for these regiments. This seems very strange, as artillery horses cost much more than cavalry horses, or even quartermasters' mules.

The veterinary profession is recognized as a learned profession by the U. S. Department of Agriculture, and by the war departments of the various civilized nations of Europe. It seems there ought to be some legislation enacted by Congress in the interest of veterinarians employed in the U. S. Army. Reputable veterinarians are not alone in entertaining the above opinion. "Calvary; Its History, Management and Uses in War," J. Roemer, L. L. D., pages 480–81, states: "In the middle ages, when the art of protecting the horse's feet was first invented, the blacksmith was horse doctor also," and in many quarters is so yet, just as in some remote rural districts the village barber is the village surgeon too, in regard to all minor operations on the human body. Veterinary surgery, which, among the ancients, was cultivated to a certain extent, was, if we except Spain, almost entirely unknown in Europe; the practice if it was left to slaves and the meanest and most ignorant farm hands. In Sweden the people looked on it as infamous; and such is the force of prejudice, that even at the present day it is still an object of contempt with many. There is no satisfactory reason, however, why the veterinarian should occupy a lower social position than any other physician. Indeed, the medical treatment of man seems less complex and less difficult of application, since it deals with only one kind of beings, endowed with the power of explaining their pains and discomforts in language; whereas among irrational animals, little can be known except from symptoms; and although the absence of moral affections and the regularity of diet greatly simplify their maladies, yet, in many cases, it is exceedingly embarrassing to determine the seat and nature of the morbid affections. But the veterinary art, after having been long despised, now begins to take rank among the most useful professions, thanks to the enlightened efforts of some governments, which have established schools for veterinary surgery, in all respects equal to other medical colleges, and similarly organized, with a regular faculty, a prescribed course, diplomas for graduates, &c. The importance of the results can hardly be overrated, if we consider how much less frequent and fatal murrain and pestilence among animals have become during the last half century; and it seems only just that men whose studies and knowledge have conferred such valuable benefits upon the community, should participate in the usual honors granted to merit. And this applies particularly to veterinary surgeons in the army, who, in most countries, still rank with non-commissioned officers, while the other surgeons, though often inferior in knowledge and skill, rank with the officers. Veterinary surgery will never fulfil the requirements of agriculture, commerce, and the army, until the profession be assimilated to that of human surgery, and until, in cavalry regiments, the veterinarian shall be no longer associated with the master tailor and boot-maker, but allowed the same privilege as officers.

